# Enhanced Mechanical and Thermal Properties of Polyimide Films Using Hydrophobic Fumed Silica Fillers

**DOI:** 10.3390/polym16020297

**Published:** 2024-01-22

**Authors:** Jongin Yeob, Sung Woo Hong, Won-Gun Koh, In Park

**Affiliations:** 1 Department of Chemical and Biomolecular Engineering, Yonsei University, 50 Yonsei-ro, Seodaemun-gu, Seoul 03722, Republic of Korea; yeopji9595@kitech.re.kr; 2 Research Institute of Clean Manufacturing System, Korea Institute of Industrial Technology (KITECH), 89 Yangdaegiro-gil, Ipjang-myeon, Seobuk-gu, Cheonan-si 31056, Republic of Korea; swhong@kitech.re.kr; 3 KITECH School, University of Science and Technology (UST), 176 Gajeong-dong, Yuseong-gu, Daejeon 34113, Republic of Korea

**Keywords:** polyimide, composites, fumed silica, mechanical property, optical property, thermal property

## Abstract

Polyimide (PI) composite films with enhanced mechanical properties were prepared by incorporating modified fumed silica (FS) particles while preserving their optical and thermal characteristics. The PI matrix was synthesized using a fluorinated diamine, a fluorinated dianhydride, and a rigid biphenyl dianhydride via chemical imidization. Commercially available FS particles, including unmodified FS particles (0-FS) and particles modified with dimethyl (2-FS), trimethyl (3-FS), octyl (8-FS), octamethylcyclotetrasiloxane (D4-FS), and polydimethylsiloxane (PDMS-FS) were used. Scanning electron microscope images and nitrogen adsorption–desorption isotherms revealed well-defined porous structures in the FS particles. The water contact angles on the composite films increased compared to those of the pristine PI films, indicating improved water resistance. The PI/0-FS films exhibited a typical trade-off relationship between tensile modulus and elongation at break, as observed in conventional composites. Owing to the poor compatibility and agglomeration of the PDMS-FS particles, the PI/PDMS-FS composite films exhibited poor mechanical performance and diminished optical characteristics. Although the longer-chained FS particles (8- and D4-FS) improved the tensile modulus of the PI film by up to 12%, a reduction of more than 20% in toughness was observed. The PI composite films containing the methylated FS particles (2- and 3-FS) outperformed 8- and D4-FS in terms of mechanical properties, with PI/3-FS films showing an over 10% increased tensile modulus (from 4.07 to 4.42 GPa) and 15% improved toughness (from 6.97 to 8.04 MJ/m^3^) at 7 wt. % silica loading. Except for the PI/PDMS-FS composites, all composite film samples exhibited more than 86% transmittance at 550 nm. Regarding thermal properties, the glass transition temperature (*T_g_*) and thermal stability remained stable for most composite films. In addition, PI/3-FS films demonstrated enhanced dimensional stability with lower coefficients of thermal expansion (from 47.3 to 34.5 ppm/°C). Overall, this study highlights the potential of incorporating specific modified FS particles to tailor the mechanical, optical, and thermal properties of PI composite films.

## 1. Introduction

Flexible electronics incorporate key components, such as flexible cells [1], flexible displays [2], flexible sensor electronics [3], and electronic skins [4], offering benefits such as flexibility, portability, wearability, and compatibility [5]. Numerous flexible substrates have been developed to achieve excellent optical properties and good thermal stability, thus fulfilling diverse application demands. Polymer materials, in particular, have attracted significant interest owing to their various adjustable advantages, including flexibility. Highly transparent polymers, such as polyethylene terephthalate (PET), polycarbonate (PC), polyarylate (PAR), polyethylene naphthalate (PEN), polyetheretherketone (PEEK), and polyethersulfone (PES), can be used as colorless flexible components. However, the optical and mechanical properties of these polymers are degraded at high processing temperatures [6].

Polyimide (PI), a high-temperature optical polymer, is the most promising candidate for flexible cover-window films [7,8]. In particular, aromatic PIs exhibit excellent thermal stability and mechanical, physical, and chemical properties, along with a low dielectric constant, high electrical insulation, low thermal expansivity, superior dimensional stability, and outstanding corrosion resistance [9,10]. Nevertheless, aromatic PIs often show strong absorption in the visible wavelength range, resulting in a dark brown or yellow color due to the formation of a charge-transfer complex (CTC) [11]. To address this issue and enable the use of PIs in flexible display devices, chemical modifications have been introduced to PI monomer structures, such as fluorine groups, bulky pendent units, alicyclic units, functional oligomers, flexible moieties, and heterocyclic groups. In particular, PI films containing fluoroalkyl groups, such as trifluoromethyl groups (–CF_3_), have shown good solubility, low dielectric constants, and extraordinary thermal and mechanical properties compared to other transparent PI matrices [12].

Organic/inorganic composites have been widely used because of the combined advantages of their two phases: organic polymers with flexibility, processability, and ductility, and inorganic fillers with rigidity, stiffness, and thermal stability [13]. In the case of PI composite films, the incorporation of inorganic fillers has been studied to enhance their mechanical and thermal properties. Inorganic materials such as metal oxides [14], organo-clays [15], boron nitride [16], carbon nanomaterials [17,18], silica nanoparticles [19,20,21,22], and polysilsesquioxane (POSS) [23,24] have been widely used to fabricate transparent PI/inorganic composite films [6]. Among these inorganic materials, silica nanoparticles offer advantages such as abundance, low cost, good adsorption, high mechanical strength, a low dielectric constant, and excellent chemical and thermal stabilities [25,26]. However, unmodified silica nanoparticles can lead to increased viscosity and a notable decrease in the glass transition temperature (*T_g_*) of the PI matrix [27] because the hydrophilic silanol groups on the silica surface promote aggregation and moisture adsorption [28]. In particular, the elongations at break (or toughness values) of the PI/silica composites were sacrificed even though the tensile moduli were improved. [19,21] Surface modification of silica nanoparticles through silylation with silane coupling agents such as alkyl, amino, acrylate, thiol, and epoxy groups has been proposed to mitigate these issues [29].

Fumed silica (FS) particles, commercially well known for their low cost, high specific surface area, rigidity, and adsorption capability, have found applications in various industries [30]. FS products are typically manufactured by spraying silicon tetrachloride in a high-temperature chamber (approximately 1500 °C) with an oxygen supply. The diameters of the primary particles were approximately 5–15 nm, and the FS nanoparticles were first formed and connected in a three-dimensional network structure. The final FS particles became nanometer-sized aggregates and micrometer agglomerates. They are white, fluffy powder-like solids exhibiting 50–300 m^2^/g of relatively high specific surface area and low bulk density (0.02–0.13 g/cm^3^) [31,32]. FS particles are used to reinforce elastomers, rheological additives, printing materials, and insulators [33]. Sarath et al. reported an improved tensile modulus for silicone rubber/FS composites at 3 wt. % unmodified FS loading but with only half the elongation of pristine rubber [34]. William et al. demonstrated cyanate ester/FS composites with two types of unmodified FS products with surface areas of 200 and 50 m^2^/g and examined the dynamic mechanical behavior of the composites [35]. Aneesa et al. introduced hydrophobic FS particles modified with hexamethyldisilzane into poly(butadiene-co-acrylonitrile) [36]. However, no reports have been published on the mechanical, optical, or thermal properties of PI composite films containing FS particles.

In this study, PI films were prepared using a diamine (2,2′-bis(trifluoromethyl)benzidine, TFDB) and two anhydrides (4,4′-(hexafluoroisopropylidene)diphthalic anhydride, 6FDA, and 3,3′,4,4′-biphenyltetracarboxylic dianhydride, BPDA). Six FS particles, one unmodified FS (0-FS) and five hydrophobic FS particles modified with dimethyl (2-FS), trimethyl (3-FS), octyl (8-FS), octamethylcyclotetrasiloxane (D4-FS), and polydimethylsiloxane (PDMS-FS), were used as inorganic fillers. Notably, the modified FS fillers are commercialized, and thus, they are readily used without surface modification. PI/FS composite films were fabricated by adding 1–10 wt. % FS particles and their mechanical, optical, and thermal properties were examined. Composite films containing relatively short-chained FS (2- and 3-FS) particles demonstrated better mechanical performance, with over a 10% increase in tensile modulus while maintaining superior toughness compared to pristine PI films at 5–7 wt. % silica loading. However, the PI/PDMS composite films exhibited poor particle dispersion, leading to a deteriorated tensile modulus and toughness, as well as low transmittance in the visible range. Despite this, other composite films demonstrated more than 86% transmittance values at a wavelength of 550 nm, along with low coefficients of thermal expansion (CTEs), while preserving *T_g_* and thermal stability.

## 2. Materials and Methods

### 2.1. Materials

**Figure 1:**
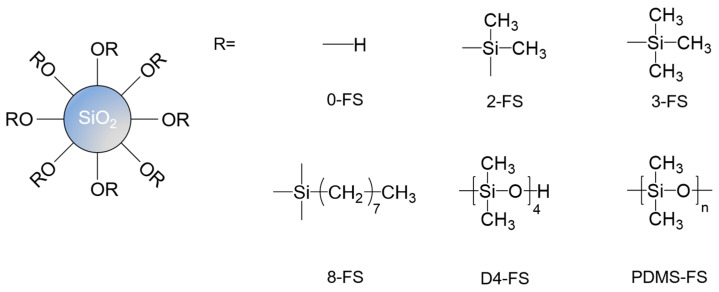
Chemical structures of the FS surfaces for the unmodified FS samples and the modified FS samples.

TFDB and 6FDA were purchased from Changzhou Sunlight Pharmaceutical Corporation (Changzhou, China), and BPDA was supplied by Shanghai Gu Chuang New Chemical Materials Corporation (Shanghai, China). Ethyl alcohol, N, N-dimethylacetamide (DMAc), acetic anhydride, and pyridine were purchased from Samchun Chemicals (Pyeongtaek, Republic of Korea). Commercially available FS powders, Aerosil 200 (0-FS), R974 (2-FS), R812S (3-FS), R805 (8-FS), R106 (D4-FS), and R202 (PDMS-FS), were supplied by Evonik (Essen, Germany), and their structures are shown in Figure 1.

### 2.2. Synthesis of PI Oligomer Powder

**Figure 2:**
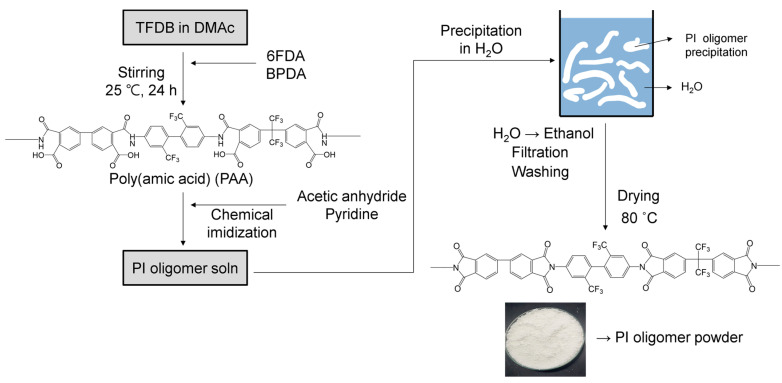
Preparation process of PI oligomer powder from PAA solution in DMAc through chemical imidization and purification using water and ethanol.

In this study, PI and PI/FS composite films were prepared via chemical imidization. The overall process is illustrated in Figure 2. Diamine and the two dianhydrides were mixed in DMAc to synthesize poly(amic acid) (PAA), and acetic anhydride and pyridine were added to the PAA solution for chemical imidization. The resulting PI oligomers were precipitated in water and washed with ethanol. Dried PI oligomer powder was obtained and dissolved in the solvent. The detailed procedure for preparing the PI oligomer powder samples is as follows.

To synthesize PAA as a PI oligomer precursor, TFDB (0.6 mol) was dissolved in 1.6 kg of DMAc at 25 °C under a nitrogen atmosphere with a mechanical stirrer. 6FDA (0.3 mol) and BPDA (0.3 mol) were added to the solution. The total monomer concentration was 20 wt. % in DMAc. After stirring for 24 h under N_2_ purging, 1.5 mol of acetic anhydride and 0.75 mol of pyridine were mixed with the PAA solution for 6 h for chemical imidization to convert PAA into PI oligomers. The reaction mixture was poured into deionized (DI) water to precipitate the PI oligomers. The white solid product was filtered and washed three times with DI water and an additional three times with ethanol. After filtering, the PI oligomer powder was dried at 80 °C in an oven (yield: 390.9 g, 94.5%).

### 2.3. Preparation of Pristine PI and PI/FS Composite Films

Chemically imidized and purified PI oligomer powder (6 g) was dissolved in DMAc (30 g) using a magnetic stirrer. After complete dissolution, the PI oligomer solution was cast onto a glass plate (borosilicate, 30 cm × 30 cm) using a film applicator. The casted solution was treated on a hot plate at 50 °C for 30 min, 150 °C for 30 min, and 250 °C for 30 min. The film was peeled off the glass plate and stored in a zipped bag. The total thickness of all films was 50 ± 2 μm. For the PI/FS composite films, 1, 3, 5, 7, and 10 wt. % of FS powders based on total weights were dispersed in DMAc in an ultrasonic bath. The FS suspension was added to the PI oligomer solution. The remainder of the process for the composite films was identical to that for the pristine film. The final PI/FS hybrid films were denoted as x-FS-y, where x-FS is the FS used as a filler for the film and y is the FS content in wt. %.

### 2.4. Characterization

Fourier transform infrared (FT-IR) spectra were obtained from 32 scans with a resolution of 8 using a Nicolet 6700 instrument (Thermo Scientific, Waltham, MA, USA). All samples were measured in the attenuated total reflection mode in the range of 4000–650 cm^−1^. The PAA powder was prepared through the precipitation of the PAA solution in DI water prior to chemical imidization. The precipitated PAA was washed with H_2_O three times and dried under a vacuum. The ultraviolet-visible (UV-vis) spectra of the PI films were recorded in the range of 300–800 nm using a Lambda 750S instrument (PerkinElmer, Waltham, MA, USA). The yellow index (YI) and haze were measured using ASTM D1003 and D1925 [37,38] spectrophotometers (CM-3600d, Konica Minolta, Tokyo, Japan). The surface morphologies of the FS particles, pristine PI film, and PI/FS composites were evaluated using a field-emission SEM (JSM-6701F, JEOL, Tokyo, Japan) at an acceleration voltage of 5 kV. All samples were sputter-coated with Pt prior to SEM measurements. Thermogravimetric analysis (TGA) measurements were conducted at a heating rate of 10 °C/min from 25 to 800 °C under nitrogen (20 mL/min) with a TGA 4000 instrument (PerkinElmer, Waltham, MA, USA). The mechanical properties of the PI films were measured using a universal testing machine (UTM, Z005, Zwick Roell, Ulm, Germany) by gripping the samples with a separation of 26.5 mm, at a rate of 10 mm/min. The specimens used for the UTM tests were die-cut into dog bone-shaped samples according to ASTM D-638 [39]. Thermomechanical analysis (TMA) measurements were performed at a heating rate of 5 °C/min from 50 to 450 °C under a nitrogen purge (20 mL/min) with a fixed tension force of 0.05 N using a TMA 4000 (PerkinElmer, Waltham, USA). Specimens for the TMA analysis were prepared with a length of 20 mm and a width of 5 mm. Dynamic mechanical analysis (DMA) was conducted using a dynamic mechanical analyzer (Q800, TA Instruments, New Castle, DE, USA) to determine the glass transition temperature (*T_g_*) of the films with tan *δ*. DMA measurements were performed in tensile film mode, at a frequency of 1 Hz and a heating rate of 5 °C/min from 25 to 450 °C. The water contact angles (CAs) were measured using the sessile drop method with an 8 μL droplet of DI water at 25 °C using a Smart Drop CA system (Femtobiomed, Seongnam, Republic of Korea). N_2_ adsorption–desorption isotherms were measured at 77 K on a Belsorp-mini II (MicrotracBEL, Osaka, Japan) after outgassing at 200 °C in a vacuum for 3 h. The specific surface areas were calculated from the adsorption branches in the range of 0.015–0.3 *P*/*P*_0_ using the Brunauer–Emmett–Teller (BET) method. The Barrett–Joyner–Halenda (BJH) theory was used to determine the pore size distributions from the adsorption branches. The pore volume was calculated at *P*/*P*_0_ = 0.95. Transmission electron microscope (TEM, JEM-2100F, JEOL, Tokyo, Japan) images and electron diffraction patterns were obtained with a TEM with an acceleration voltage of 200 kV. The specimens were prepared with a thickness of 100 nm using an ultramicrotome (Powertome, RMC, Tucson, AZ, USA).

## 3. Results and Discussion

### 3.1. Characterizations of FS Particles

**Figure 3:**
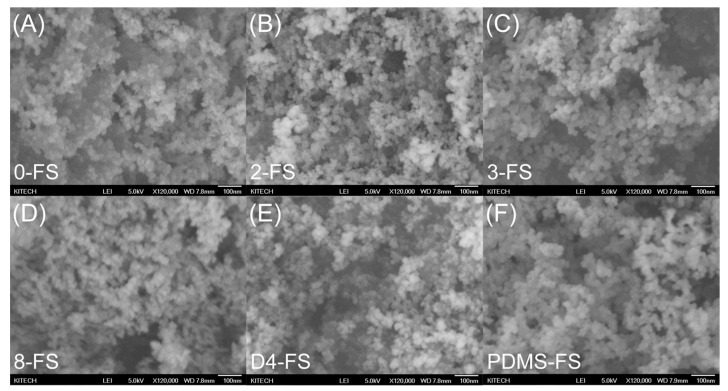
SEM images of FS particles: (**A**) 0-FS, (**B**) 2-FS, (**C**) 3-FS, (**D**) 8-FS, (**E**) D4-FS, and (**F**) PDMS-FS (the scale bar on the bottom right is 100 nm).

Six types of FS particles were used to fabricate the PI/FS hybrid films. One unmodified FS particle (0-FS) and five modified FS particles—dimethyl (2-FS), trimethyl (3-FS), octyl (8-FS), octamethylcyclotetrasiloxane (D4-FS), and polydimethylsiloxane (PDMS-FS)—were used. The SEM images in Figure 3 show the surface morphologies of the FS particles. All types of modified FS exhibited a particle morphology similar to that of the 0-FS particles, consisting of three-dimensional networks of approximately 25 nm primary particles. Irregular FS pores formed by the interconnected primary particles were observed with sizes of 10–150 nm, with them belonging to the meso- or macropore range [40,41].

**Figure 4:**
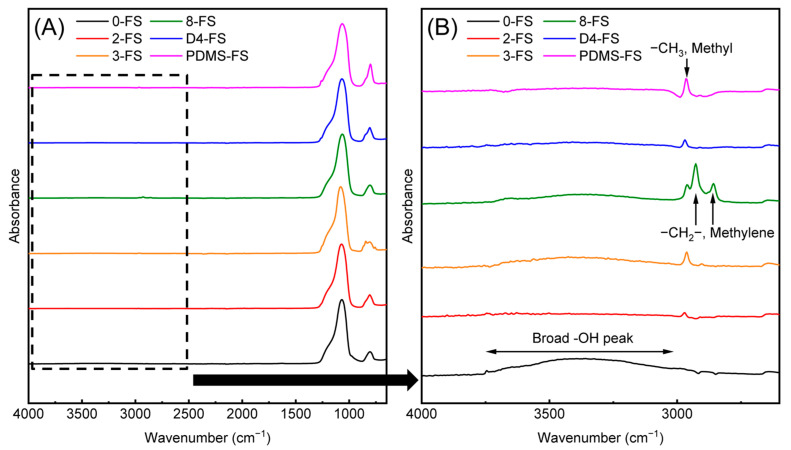
FT-IR spectra of the FS materials in the range of (**A**) 450–4000 cm^−1^ and (**B**) 2600–3500 cm^−1^ to clearly show the –CH_2_ and –CH_3_ stretching bands.

The FTIR spectra of the FS particles are shown in Figure 4. All of the FS particles exhibited Si–O–Si vibration peaks at 470, 810, and 1109 cm^−1^, Si–OH stretching at 1640 cm^−1^, and a broad peak of O–H absorbance at approximately 3500 cm^−1^. 0-FS exhibited the largest broad peaks of Si–OH and O–H, indicating a large number of silanol groups and absorbed water on the silica surface. For the modified FS particles, the intensities of the O–H, and Si–OH absorption bands decreased because the surface of the FS was modified with organic modifiers. Asymmetric stretching of methyl (–CH_3_) was observed at 2960 cm^−1^ for the 2-, 3-, D4-, and PDMS-FS materials. For 8-FS, the asymmetric and symmetric peaks of methylene (–CH_2_) were observed at 2930 and 2860 cm^−1^, respectively.

**Figure 5:**
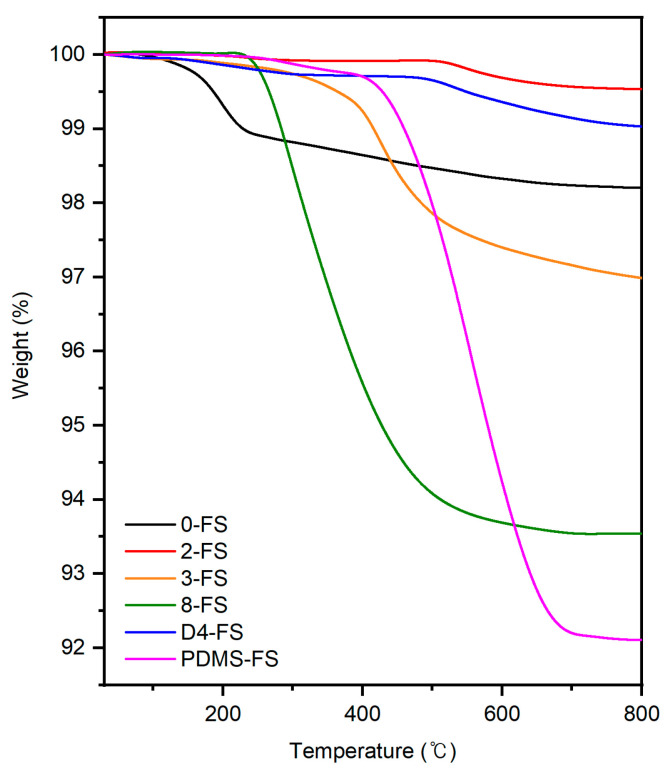
TGA curves of the FS particles.

TGA curves of the FS materials (Figure 5) were obtained to investigate the grafting ratios of the modifiers listed in Table 1. The hydrophilic FS, 0-FS, showed that the evaporation of adsorbed moisture started at 100 °C and ended at approximately 200 °C. Derivative thermogravimetric (DTG) analysis on metal oxides modified with long-chain hydrocarbons showed that the long-chain hydrocarbon chain decomposes thermally at approximately 250 °C (maximum temperature in a DTG curve) and the methyl chain decomposes thermally at approximately 450 °C [42], matching the TGA results for the 2-, 3-, and 8-FS in Figure 5. D4- and PDMS-FS exhibited a maximum temperature of approximately 550 °C because the relatively high bond strength of Si–O–Si remains on the surface of the fumed silica. Graft ratios are listed in Table 1. A slightly lower surface grafting ratio (or carbon content) was observed for the 2- and D4-FS samples than that for the samples provided by Evonik, the manufacturer of the FS materials.

The N_2_ adsorption–desorption isotherms (Figure S1) of the FS particles were measured, and the BET-specific surface areas and pore sizes are listed in Table 1. All of the isotherms belong to type II, which represents a typical nonporous or macroporous solid according to the IUPAC classification. However, the FS pores formed by the three-dimensional network structure of silica nanoparticles were widely distributed in the meso- and macro-range, as observed in the SEM images of the FS materials. Despite the peaks at 14–20 nm in the pore-size distributions of the samples, the N_2_ adsorption isotherms were generally limited to the mesopore range. In the same manner, the pore volumes in Table 1 calculated at *P*/*P*_0_ = 0.95 do not represent all of the FS pores. Moreover, there are many grades of commercialized FS (unmodified), and modified FS products are produced for special purposes. Therefore, a direct comparison between FS particles may not be quite effective, but the basic and relative properties of the samples can be compared. In addition, the porosity data from the isotherms are important for explaining the resulting properties of composite PI films containing FS particles. 8-FS and PDMS-FS with relatively long-chain modifiers exhibited relatively low surface areas, pore volumes, and pore sizes. Moreover, the modifier grafting ratios of the FS particles are higher than those of the other modified FS particles. The chain length of D4 was similar to that of the octyl modifier of 8-FS, but the D4 modification ratio was relatively low, resulting in a higher surface area, pore volume, and pore size than those of 8-FS. The 2- and 3-FS samples were modified with relatively short chains, resulting in larger pore sizes than those of the other modified samples. The 2-FS material exhibits a low grafting ratio, surface area, and pore volume. As mentioned above, there are many grades of unmodified FS, and each modified FS may be prepared from a different mother FS.

### 3.2. Preparation of PI Films

**Figure 6:**
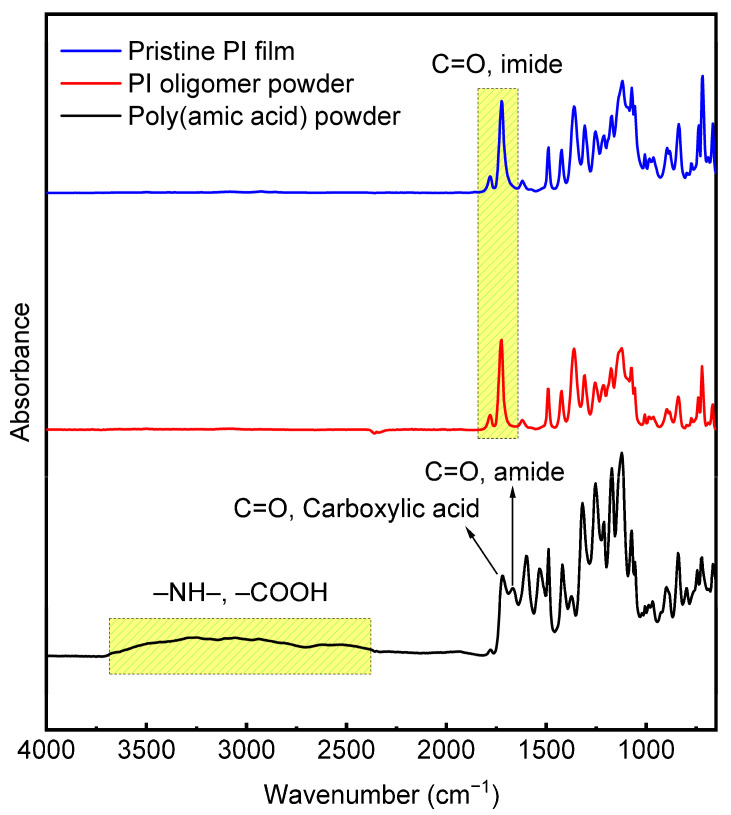
FT-IR spectra of the PAA powder, the PI oligomer powder, and the pristine PI film.

One type of diamine (TFDB) and two types of dianhydride monomers (6FDA and BPDA) were used to prepare PI films with equimolar amounts of diamine and dianhydride (TFDB:BPDA:6FDA = 100:50:50). Because fluorinated PI monomers (TFDB and 6FDA) degrade the mechanical and thermal properties of PI films, non-fluorinated biphenyl BPDA was introduced to overcome the disadvantages attributed to fluorinated monomers. In the chemical imidization method, PI was prepared using typical two-step reactions, including ring-opening polyaddition for PAA and a subsequent cyclic dehydration reaction for the final product, PI. The polyaddition reaction was accomplished through the use of a mechanical stirrer at 25 °C for 24 h and a viscous and transparent PAA solution was obtained. A few grams of PAA solid powder were obtained from an IR sample through drying of the solvent. The chemical imidization of PAA was then performed by adding a dehydrating agent (acetic anhydride) and a catalyst (pyridine). The PI oligomer solution was precipitated with DI water and washed with ethanol. The dried solid PI oligomer product was ground and dissolved in DMAc solvent. The final pristine PI film was obtained by casting it on a glass plate and subjecting it to heat treatment. Figure 6 shows the FTIR spectra of the PAA powder, the PI oligomer powder, and the pristine PI film. The peaks at 1720 and 1670 cm^−1^ correspond to the C=O stretching bands of carboxylic acid and amide groups, respectively, and the absorption peaks near 2500–3500 cm^−1^ are correlated with the –NH– and –COOH of amic acid in the PAA powder. After the chemical imidization of PAA, all of the peaks related to amic acid disappeared, and the peaks associated with C=O imide stretching bands at 1780 (asymmetric) and 1725 cm^−1^ (symmetric stretching) were detected for the PI oligomer powder and the pristine PI film, along with the C–N stretching vibration of the imide ring at 1361 cm^−1^. Absorption peaks near 1110–1310 cm^−1^ corresponding to the C–F group stretching from the fluorinated monomers were observed for all samples, as shown in Figure 6.

PI/FS composite films containing FS particles (0-, 2-, 3-, 8-, D4-, and PDMS-FS) with different loadings (1, 3, 5, 7, and 10 wt. %) were prepared. Figure S2 shows the FT-IR spectra of the pristine PI and the PI/FS composite films. Silica absorption peaks at approximately 1056–172 cm^−1^ (Si–O–Si asymmetric stretching), 805 cm^−1^ (Si–O–Si symmetric stretching), and 965 cm^−1^ (Si–OH stretching) were observed for the PI/FS hybrid films, and the adsorption peak intensity increased gradually as the silica content increased for all composite samples. Similarly, the residual amounts of silica particles in the TGA curves (Figure S3) of the composite films increased with increasing FS content.

**Figure 7:**
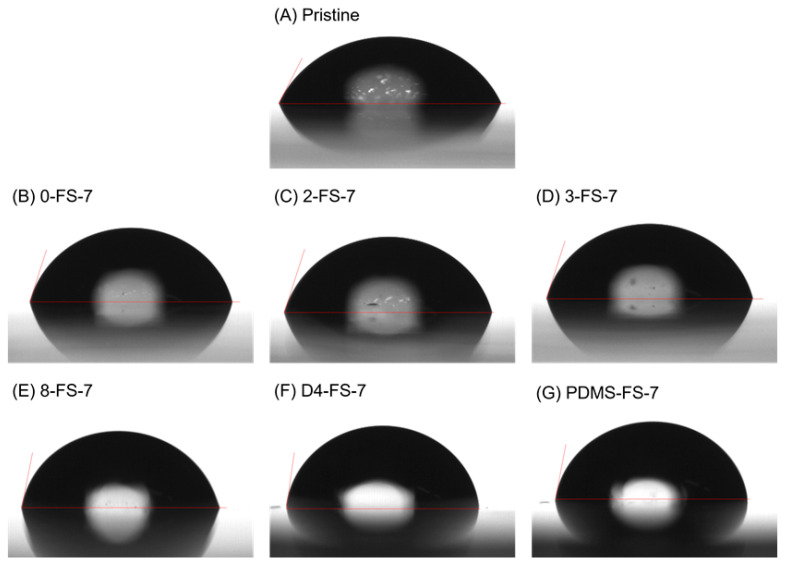
Digital photo images of the water droplets on (**A**) the pristine PI and the PI/FS composite films containing (**B**) 0-, (**C**) 2-, (**D**) 3-, (**E**) 8-, (**F**) D4- and (**G**) PDMS-FS-7.

The water CAs of the pristine PI and PI/FS composite films containing 7 wt. % FS were measured to examine the hydrophobicity of the film surfaces (Table 2, Figure 7) because porous silica materials may boost the accessibility of outside liquid water into the PI composite films. The PI/FS composite films containing 7 wt. % samples were chosen because the particles are located close to each other (see TEM images in Figure S4) enough to generate surface roughness. The surface tension of a flat PI film is known to be approximately 40 mN/m [43]. The CA of the pristine PI film was 65.2°, similar to that of the PET and polyvinyl chloride (PVC) films [44]. Hydrophobic flat polymer surfaces (Young’s model), such as PI, PET, and PVC, exhibited a higher water CA when the surfaces were rough, according to the Wenzel [45] and Cassie [46] models [47]. The PI film containing 7 wt. % 0-FS exhibited a CA of 74.9°, which was higher than that of the pristine film. The hydrophilic FS was not exposed to the surface, and the PI polymer chains covered the FS particles so that the surface of the 0-FS-7 film was rougher than that of the pristine film. The CAs of 2-FS-7 and 3-FS-7 were similar to those of 0-FS-7 because the methyl groups on the surfaces of the FS particles were too short to lower the surface tension of the composite films. The PI/FS composite films containing relatively long-chain alkyl or dimethylsiloxanes exhibited higher water CAs because the chains were long enough to be exposed to the air side of the films, lowering the surface energy of the film surfaces and generating more roughness.

### 3.3. Mechanical Properties of the Films

**Figure 8:**
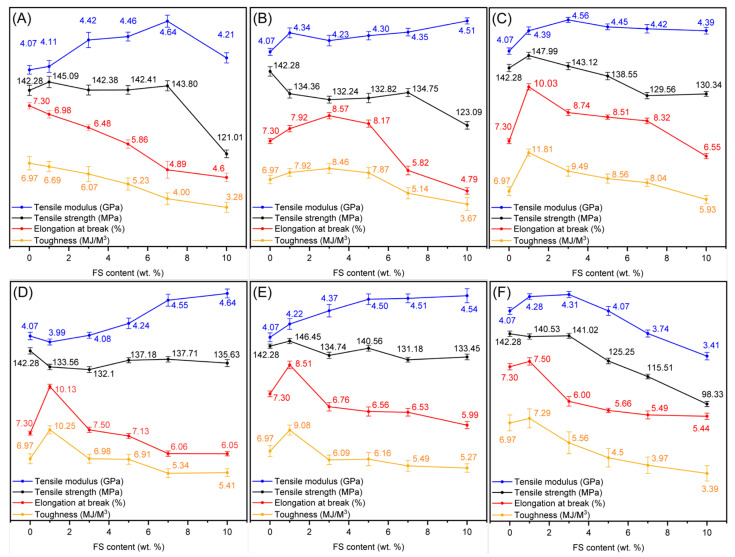
Tensile properties of the pristine PI and the PI/FS composite films. Tensile moduli (blue), tensile strength (black), elongations at brake (red), and toughness (orange) of the PI composite films containing (**A**) 0-, (**B**) 2-, (**C**) 3-, (**D**) 8-, (**E**) D4-, and (**F**) PDMS-FS as a function of FS loadings.

Figure 8 shows the tensile properties of the pristine PI and PI/FS composite films used to investigate the effects of hydrophilic and hydrophobic FS particles on the mechanical (tensile) properties of the PI films. Typically, polymer composites with rigid inorganic fillers show an increase in tensile modulus and strength, but a decrease in elongation at break and toughness as the content of the inorganic filler is increased [48]. The PI/FS composite films with 0-FS show a common trade-off relationship between modulus and elongation-at-break, as mentioned above. The tensile strength and tensile modulus increased up to the 0-FS-7 composite film, exhibiting the highest tensile modulus of approximately 14%; however, the elongation at break and toughness of the 0-FS hybrid films decreased gradually compared to that of the pristine film. Although the PI/FS composite film containing flexible and elastomeric long PDMS exhibited higher values of modulus and toughness at a low (1 wt. %) FS loading, undesirable results were observed at higher PDMS-FS loadings. Hydrophobic FS materials with similar surface modifier chain lengths, 8- and D4-FS, had similar effects on the tensile properties of the PI composite films. Notably, the PI/FS composite films containing FS modified with short chains (methyl) exhibited higher toughness values and moduli over the range of 1–5 wt. % silica loadings than those of the pristine PI film. In particular, 3-FS particles enhanced the tensile properties of the PI films more than the other fillers. For the PI films with 3-FS, the toughness was not significantly compromised, and the modulus was still enhanced relative to that of the pristine PI film. Similar to the 3-FS-x samples, the PI/FS composite films containing the 2-FS particles possessed higher moduli and toughness than those of the pristine film; however, the effect of the particles on the mechanical performance was not as significant as that of the 3-FS films. This was due to the relatively low grafting ratio (approximately 0.4 wt. %) of the dimethyl groups on the 2-FS surfaces. Consequently, the relatively long chained modifier has a positive effect on the tensile modulus at high loading levels as the values are 4.64 (14%) and 4.54 (12% increment) GPa for 8-FS-10 and D4-FS-10. However, the toughness of the films decreased by more than 10%. Conversely, the 2-FS-x and 3-FS-x films exhibited higher tensile moduli and toughness values than the pristine film in the range of 1–5 and 1–7 wt. %, respectively.

### 3.4. Optical Properties of the Films

**Figure 9:**
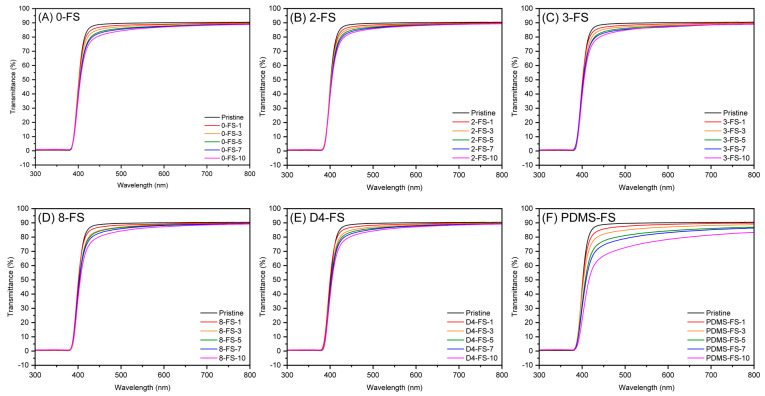
UV-vis spectra of the pristine PI and PI/FS composite films: (**A**) 0-, (**B**) 2-, (**C**) 3-, (**D**) 8-, (**E**) D4-, and (**F**) PDMS-FS.

**Figure 10:**
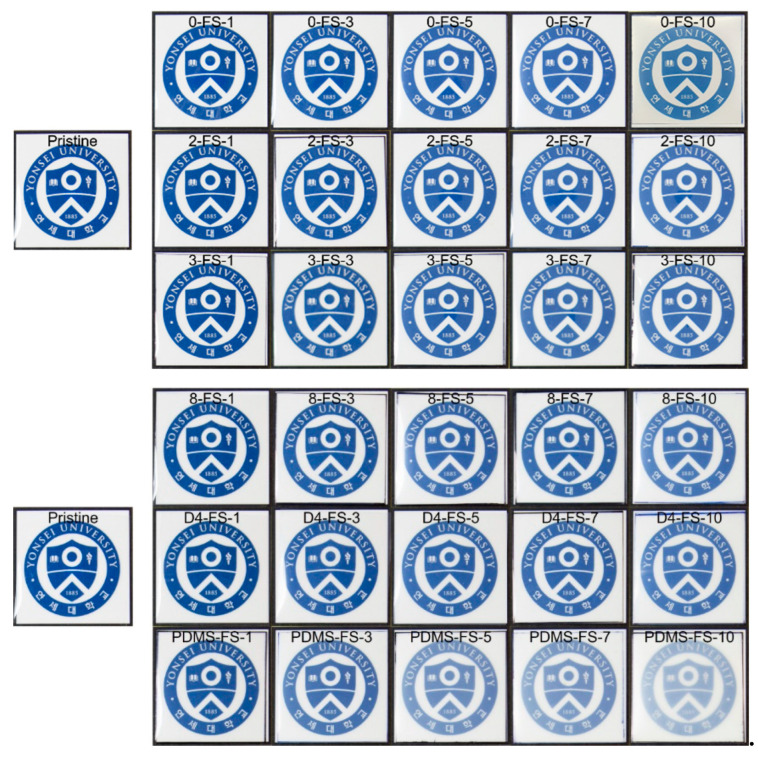
Optical images of the pristine and PI/FS composite films.

The UV-vis spectra of the PI films and optical images are shown in Figure 9 and Figure 10, respectively. The transmittances at 550 nm (*T*_550_), YI, and haze are listed in Table 3. Generally, PI films prepared from fluorinated monomers are optically transparent because the CF_3_ groups in TFDB and 6FDA hinder charge–transfer interactions between the donor and acceptor complexes. The transmittance of the pristine PI film was 89.9% at 550 nm and it exhibited low YI and haze values, indicating its transparency. The PDMS-FS-x films with >1wt. % FS content were more opaque (Figure 10) than the other PI/FS composite films, as observed from the optical images. The transmittance of PDMS-FS-5 was approximately 83%, indicating the low dispersibility of the PDMS-FS particles due to the incompatibility between the PI and PDMS polymer chains [49,50]. In particular, their high haze values were due to the agglomeration of the PDMS-FS particles, as shown by the deteriorated tensile properties in Figure 8. For the other composite films, all of the *T*_550_ values were >86% up to 10 wt. % loading, independent of the modifiers. However, the YI and haze values tended to deviate from the transmittance. The modified FS fillers, 2-, 3-, 8-, and D4-FS exhibited almost identical increments in the YI values of as much as 7–9 at 7–10 wt. % loadings, but the YI values of the unmodified FS were relatively lower than those of the other composite films. This indicated that the YIs of the composite films were affected by the modifiers. However, the haze values of the 0-FS-x composite films were higher than those of the PI/FS composite films. The 0-FS particles agglomerated because of hydrogen bonding between the surface silanol groups, which increased the haze values through strong light scattering. Moreover, the –OH groups of the 0-FS surface were difficult to disperse in the organic matrix compared with the hydrophobic FS surfaces, as predicted by the inferior tensile characteristics of the PI/FS composite films containing 0-FS.

The optical images of the pristine and PI/FS composite films are shown in Figure 10. The PI/PDMS-FS composite films with relatively low transmittance and high haze values were more blurred than the other films at the same FS loading levels. No optical yellowness was detected in the optical images of any of the samples. However, the color of the composite became milkier with increasing FS content, as predicted by the haze index. Although it is not easy to explain the relationship between the aggregation of the FS particles and the cross-sectional SEM images of the film samples in Figure S5, more agglomeration of the FS particles was observed as the FS loading increased for all composite films. Notably, agglomerates of PDMS-FS particles were observed in the film, even at a silica loading of 1 wt. % (Figure S5). In contrast, relatively clear cross-sectional images were obtained for the other composite films at low silica loadings (1–5 wt. %).

### 3.5. Thermal Properties of Films

The thermal properties of the PI films (Table 4) were examined by means of TGA (Figure S3), DMA, and TMA (Figure S6). The thermal decomposition temperature (*T_d_*) at 5% weight loss in the TGA curves is denoted as *T_d_*_5%_. As the polymer-based composites containing porous fillers exhibited retarded thermal degradation [51], enhanced thermal stabilities of 1–2% were observed based on the *T_d_*_5%_ values in the PI/FS composite films. To verify the silica amount of the films, the residual amounts were calculated using the following equation: 0.429 × (100 − *φ*) + *φ* × (1 − *r*/100), where 0.429 is the residual amount of the pure PI, *φ* is the weight percent of the FS filler, and *r* is the grafting ratio of the organic modifier. The differences between the experimental and calculated residual amounts of the composite films at 800 °C were within 4%, while those of PI/PDMS composites were up to 8% owing to the long PDMS chains and the relatively high grafting ratio. The glass transition temperatures (*T_g_*) of the composites were determined from the peaks of tan *δ* (Figure S7) from the DMA analysis. The highest *T_g_* values for the composite films were within ±3% difference from that of the pristine film, indicating no or little notable change in *T_g_*. 

To compare the thermally dimensional stability of PI/FS composite films, Table 4 shows CTE values for the PI/FS composite films as a function of the silica contents calculated from the slope from 50 to 200 °C in the TMA curves (Figure S6). Typically, the CTE of silica is only 0.5 ppm/°C; therefore, the composite films exhibited lowered CTEs because the stiffness and rigidity of FS particles and the interaction between the PI matrix and FS were hindered by the movement of the composite chain [52]. Thus, the composites showed typical characteristics of PI/SiO_2_ composites, which lowered the CTEs with increasing silica content, as indicated by the improved tensile moduli. Owing to the rigidity of the unmodified 0-FS and 2-FS samples with low grafting ratios, their composite films exhibited relatively low CTEs compared to the other composite films. Films containing 8- and PDMS-FS with long-chain modifiers and high grafting ratios showed higher CTEs at high silica loadings than those with lower silica contents. In the case of the PI/3-FS composite films exhibiting relatively high mechanical performance, CTE drops of >20% were observed at 7 and 10 wt. % silica loadings.

## 4. Conclusions

In this study, PI composite films were fabricated by incorporating modified FS particles to enhance their mechanical properties without compromising their optical and thermal characteristics. Commercially available FS particles, including unmodified FS particles (0-FS) and modified with dimethyl (2-FS), trimethyl (3-FS), octyl (8-FS), octamethylcyclotetrasiloxane (D4-FS), and polydimethylsiloxane (PDMS-FS), were used so that the modified FS fillers could be readily used in industrial fields. The modifiers were verified using FT-IR spectra for all modified FS particles, although the grafting ratios were relatively low for the 2-FS and D4-FS particles. The water CAs of the PI composite films containing methylated FS particles (2- and 3-FS) were approximately 10° higher than those of the pristine PI film, indicating improved water resistance. The mechanical performance of the PI/0-FS composite films demonstrated typical behavior associated with conventional inorganic fillers, exhibiting a tradeoff relationship between the tensile modulus and elongation at break. Because of the poor compatibility and dispersibility of the long dimethylsiloxane chains, the PI composite films containing PDMS-FS exhibited poor mechanical properties, including both modulus and elongation. The diminished optical properties of the films, such as haze and transmittance, were also attributed to the low compatibility and agglomeration of the PDMS-FS particles. In comparison, shorter-chained FS particles (2- and 3-FS) outperformed 8- and D4-FS in terms of mechanical properties, as evidenced by their improved tensile modulus and toughness. Specifically, the PI/3-FS composite film with silica loading of 7 wt. % was the optimum condition, with it demonstrating a >10% increase in the tensile modulus and better toughness by 15% than the pristine PI film. Moreover, except for the PI/PDMS-FS films, no critical reduction in the transmittance in the visible range or *T_g_* values was observed for the composite films. Additionally, enhanced dimensional stability was observed in the PI/3-FS composite films, as indicated by the lower CTEs, even though the PI composite films containing unmodified FS had lower CTEs at the same silica loadings.

## Figures and Tables

**Table 1:** Surface modifier ratios calculated from the TGA curves and physicochemical properties of the FS samples calculated from the N_2_ adsorption–desorption isotherms.

Sample	Surface Grafting Ratio (wt. %)	BET Surface Area (m^2^/g)	Pore Size (nm)	Pore Volume (cm^3^/g)
0-FS	0	200	14.0	0.83
2-FS	0.4	135	19.4	0.84
3-FS	3.0	221	20.6	1.45
8-FS	6.5	106	14.4	0.60
D4-FS	0.75	243	19.5	1.48
PDMS-FS	7.9	105	15.8	0.62

**Table 2:** Water CAs on the pristine PI and the PI/FS composite films.

Sample	Water CA (°)
Pristine	65.2
0-FS-7	74.9
2-FS-7	74.6
3-FS-7	75.0
8-FS-7	80.5
D4-FS-7	81.3
PDMS-FS-7	81.7

**Table 3:** Optical properties of the pristine and PI/FS composite films.

Sample	*T*_550_ *	YI	Haze
Pristine	89.9	4.0	0.26
0-FS-1	88.7	4.6	3.57
0-FS-3	88.0	5.3	6.61
0-FS-5	87.1	6.3	11.67
0-FS-7	86.7	7.3	13.62
0-FS-10	86.0	6.3	29.04
2-FS-1	88.9	4.5	1.69
2-FS-3	88.3	5.6	5.60
2-FS-5	87.8	6.8	9.19
2-FS-7	87.5	7.2	11.41
2-FS-10	87.1	9.0	15.60
3-FS-1	88.8	4.8	2.85
3-FS-3	87.8	5.4	7.95
3-FS-5	87.1	6.6	13.35
3-FS-7	86.4	7.0	17.20
3-FS-10	86.5	8.4	17.95
8-FS-1	88.9	4.6	4.04
8-FS-3	88.1	6.6	10.77
8-FS-5	87.8	6.8	14.76
8-FS-7	87.3	7.9	15.20
8-FS-10	86.3	9.5	18.30
D4-FS-1	88.9	4.5	3.27
D4-FS-3	87.9	6.1	8.12
D4-FS-5	87.3	6.8	10.03
D4-FS-7	86.9	7.7	13.10
D4-FS-10	86.2	9.0	14.43
PDMS-FS-1	88.4	5.7	11.65
PDMS-FS-3	86.3	6.7	24.95
PDMS-FS-5	83.0	8.4	46.50
PDMS-FS-7	81.5	10.0	50.39
PDMS-FS-10	76.1	13.2	67.71

* Transmittance at 550 nm.

**Table 4:** Thermal properties of the pristine and PI/FS composites.

	TGA			DMA		TMA
	*T_d_*_5*%*_ (°C)	Residue Amount (wt. %)		tan *δ*	*T_g_* (°C)	CTE (ppm/°C)
		Experimental	Calculated			
Pristine	528.3	42.9	N/A	0.65	360.8	47.3
0-FS-1	534.5	44.6	43.5	0.72	358.0	39.7
0-FS-3	534.1	46.0	44.6	0.68	360.2	39.6
0-FS-5	536.1	46.1	45.8	0.63	370.3	38.2
0-FS-7	535.2	47.8	46.9	0.64	367.3	37.2
0-FS-10	537.7	50.0	48.5	0.56	366.2	28.9
2-FS-1	534.7	44.9	43.5	0.71	367.1	39.9
2-FS-3	534.8	45.5	44.6	0.71	366.6	37.0
2-FS-5	534.6	46.4	45.7	0.70	364.0	40.1
2-FS-7	531.6	48.6	46.9	0.63	357.7	35.9
2-FS-10	534.4	50.4	48.6	0.62	357.8	30.4
3-FS-1	531.3	44.8	43.4	0.68	367.9	37.4
3-FS-3	532.5	45.7	44.5	0.69	359.0	34.0
3-FS-5	530.4	47.4	45.6	0.69	368.5	38.4
3-FS-7	533.0	48.3	46.7	0.60	367.1	34.5
3-FS-10	531.3	49.8	48.3	0.61	370.1	36.9
8-FS-1	529.1	43.8	43.4	0.61	361.2	37.1
8-FS-3	532.4	45.6	44.4	0.65	355.7	37.5
8-FS-5	536.9	46.4	45.4	0.62	369.9	29.3
8-FS-7	532.6	47.4	46.4	0.63	357.4	39.8
8-FS-10	531.2	49.8	48.0	0.63	357.1	42.0
D4-FS-1	534.5	44.6	43.5	0.70	363.4	45.9
D4-FS-3	528.7	45.7	44.6	0.66	361.4	40.6
D4-FS-5	530.3	47.2	45.7	0.61	356.1	38.2
D4-FS-7	533.3	48.7	46.9	0.61	365.0	38.7
D4-FS-10	532.1	48.3	48.5	0.58	357.2	36.7
PDMS-FS-1	532.8	44.9	43.4	0.68	361.3	43.1
PDMS-FS-3	533.8	47.2	44.4	0.70	361.6	41.4
PDMS-FS-5	531.5	48.8	45.4	0.60	365.6	36.9
PDMS-FS-7	536.9	50.4	46.3	0.66	359.4	36.5
PDMS-FS-10	537.6	51.1	47.8	0.60	354.7	42.0

## Data Availability

Data are contained within the article and Supplementary Materials.

## Supplementary Materials

The following supporting information can be downloaded at: https://www.mdpi.com/article/10.3390/polym16020297/s1. Figure S1: N_2_ adsorption–desorption isotherms of FS nanoparticles used for PI/FS composite films: (A) 0-, (B) 2-, (C) 3-, (D) 8-, (E) D4-, and (F) PDMS-FS; Figure S2: FT-IR spectra of the pristine PI and the PI/FS composite film containing (A) 0-, (B) 2-, (C) 3-, (D) 8-, (E) D4-, and (F) PDMS-FS. The FT-IR spectra were normalized based on the intensity of the C=O stretching peak (1670 cm^−1^) for comparison; Figure S3: TGA curves of the PI/FS composite films containing (A) 0-, (B) 2-, (C) 3-, (D) 8-, (E) D4-, and (F) PDMS-FS; Figure S4: TEM images of (A) the pristine, (B) 3-FS-1, and (C) 3-FS-7. (D–F) are the electron diffraction patterns corresponding to (A–C); Figure S5: Cross-sectional FE-SEM images of PI/FS composite films.; Figure S6: TMA curves of the PI/FS composite films containing (A) 0-, (B) 2-, (C) 3-, (D) 8-, (E) D4-, and (F) PDMS-FS; Figure S7: Tan *δ* curves of the PI/FS composite films containing (A) 0-, (B) 2-, (C) 3-, (D) 8-, (E) D4-, and (F) PDMS-FS.
