# Supplementary material for: Enhanced Mechanical and Thermal Properties of Polyimide Films Using Hydrophobic Fumed Silica Fillers
Source: Polymers (Basel). 2024 Jan 22;16(2):297. doi: 10.3390/polym16020297 (PMC10820428; doi:10.3390/polym16020297)
Supplement: Supplementary file 1 [file polymers-16-00297-s001.zip › polymers-2804717-supplementary.pdf]

Supporting Information

Enhanced Mechanical and Thermal Properties of Polyimide Films Using Hydrophobic Fumed Silica Fillers

Jongin Yeob[1,2], Sung Woo Hong[2], Won-Gun Koh[1,*] and In Park[2,3,*]

[1] Department of Chemical and Biomolecular Engineering, Yonsei University, 50 Yonsei-ro Seodaemun-gu, Seoul 03722, Republic of Korea

[2] Research Institute of Clean Manufacturing System, Korea Institute of Industrial Technology (KITECH), 89 Yangdaegiro-gil, Ipjang-myeon, Seobuk-gu, Cheonan-si, Chungnam 31056, Republic of Korea; yeopji9595@kitech.re.kr (J.Y.)

[3] KITECH School, University of Science and Technology (UST), 176 Gajeong-dong, Yuseong-gu, Daejeon 34113, Republic of Korea

[*] Corresponding author: Email address: wongun@yonsei.ac.kr (W.G.K.), inpark@kitech.re.kr (I.P.)

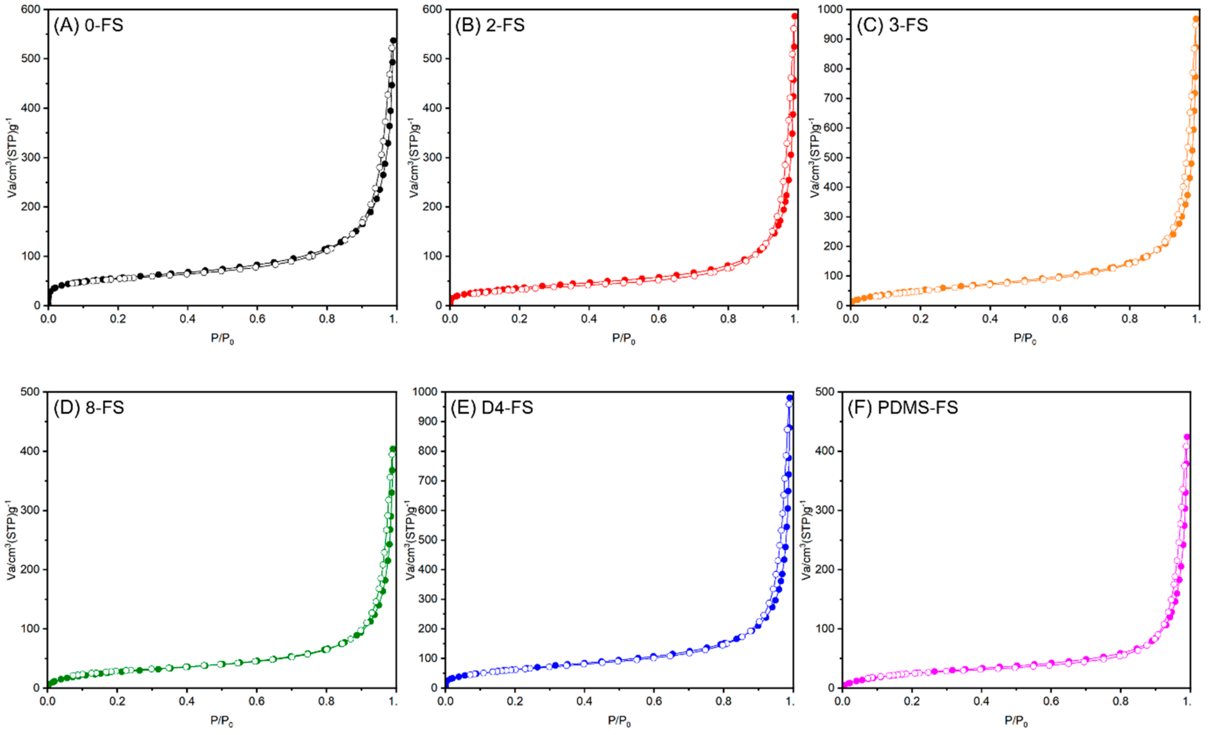

Figure S1. N_2 adsorption-desorption isotherms of FS nanoparticles used for PI/FS composite films; (A) 0-, (B) 2-, (C) 3-, (D) 8-, (E) D4-, and (F) PDMS-FS.

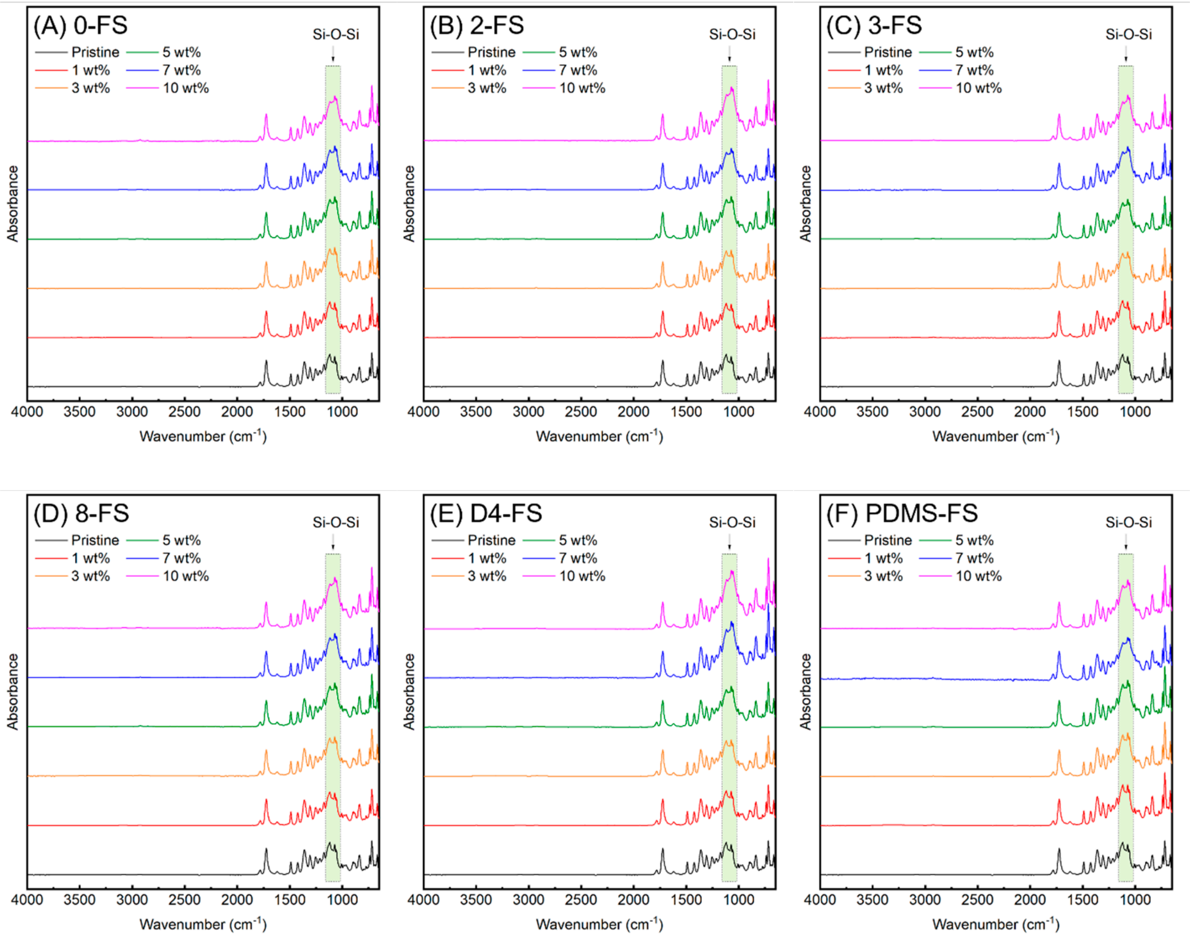

Figure S2. FT-IR spectra of the pristine PI and the PI/FS composite film containing (A) 0-, (B) 2-, (C) 3-, (D) 8-, (E) D4-, and (F) PDMS-FS. The FT-IR spectra were normalized based on the intensity of C=O stretching peak (1670 cm^{-1}) for comparison.

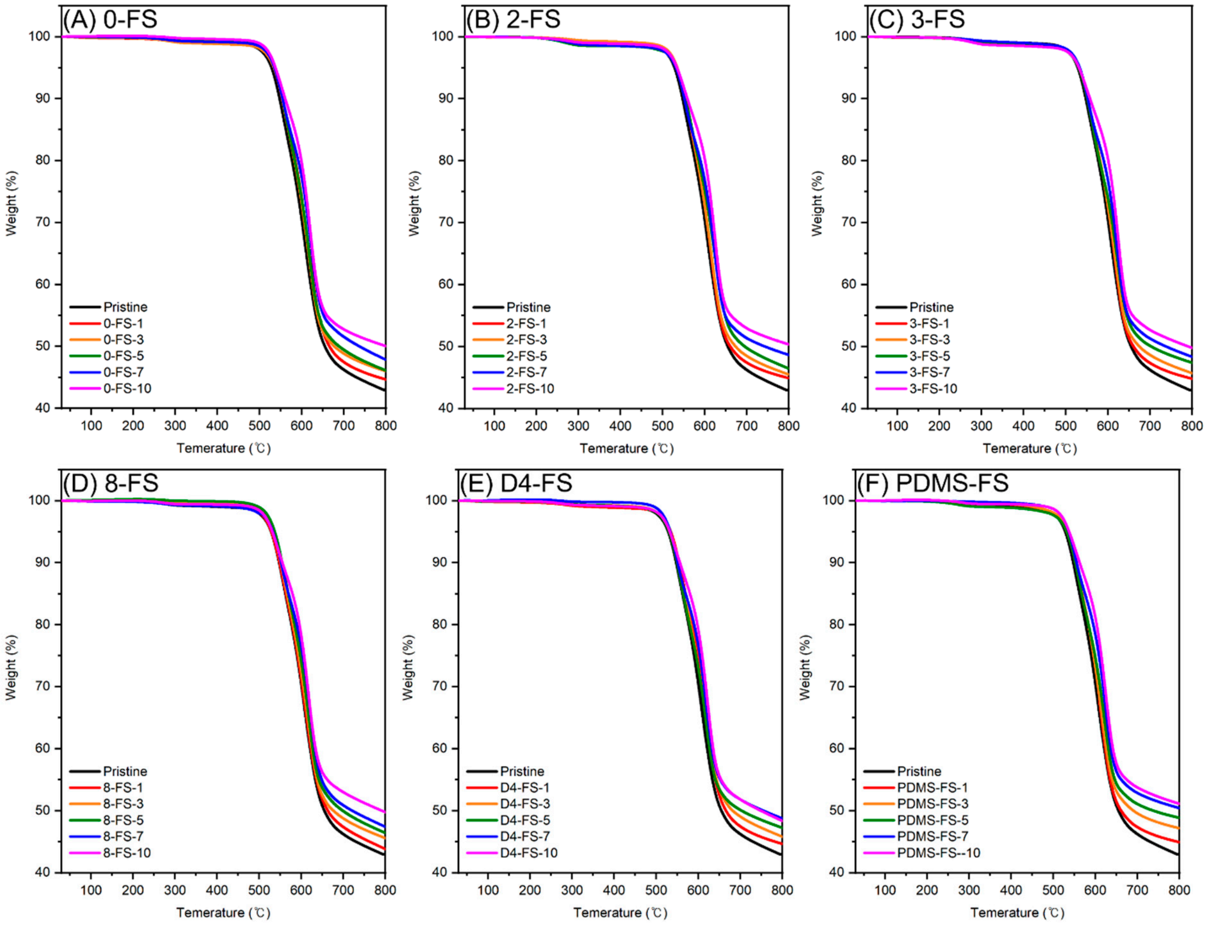

Figure S3. TGA curves of the PI/FS composite films containing (A) 0-, (B) 2-, (C) 3-, (D) 8-, (E) D4-, and (F) PDMS-FS.

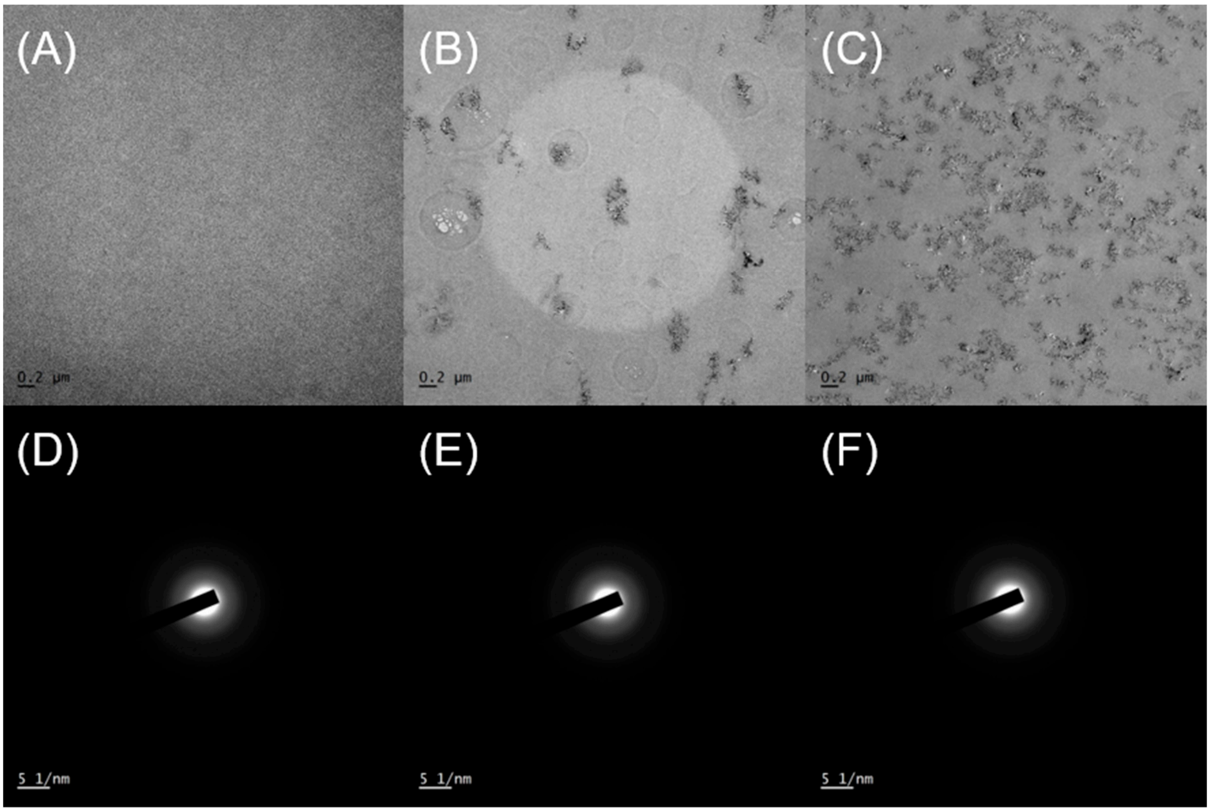

Figure S4. TEM images of (A) the pristine, (B) 3-FS-1, and (C) 3-FS-7. (D), (E), and (F) are the corresponding electron diffraction patterns to (A), (B), and (C).

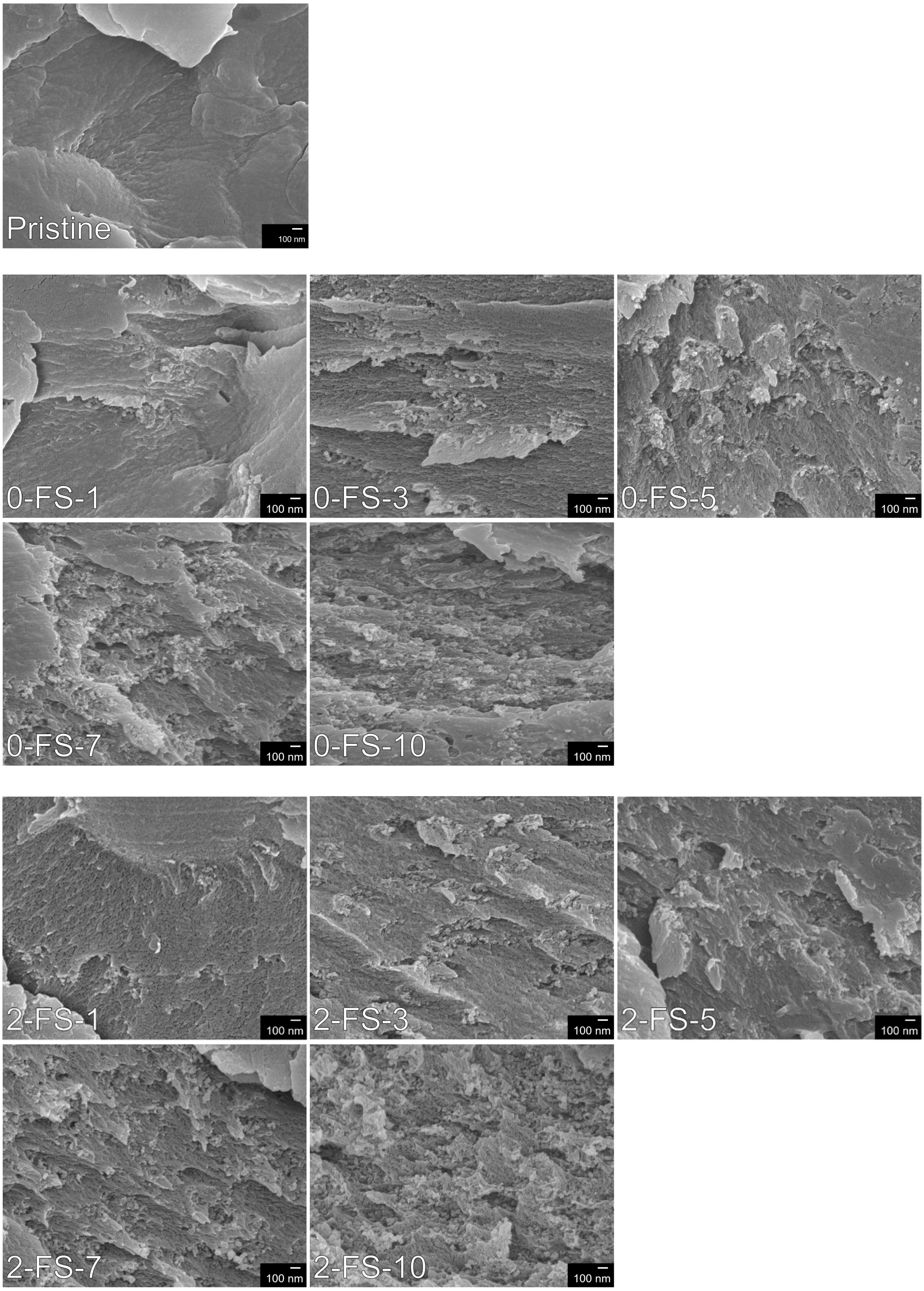
Pristine
100 nm
0-FS-1
100 nm
0-FS-3
100 nm
0-FS-5
100 nm
0-FS-7
100 nm
0-FS-10
100 nm
2-FS-1
100 nm
2-FS-3
100 nm
2-FS-5
100 nm
2-FS-7
100 nm
2-FS-10
100 nm

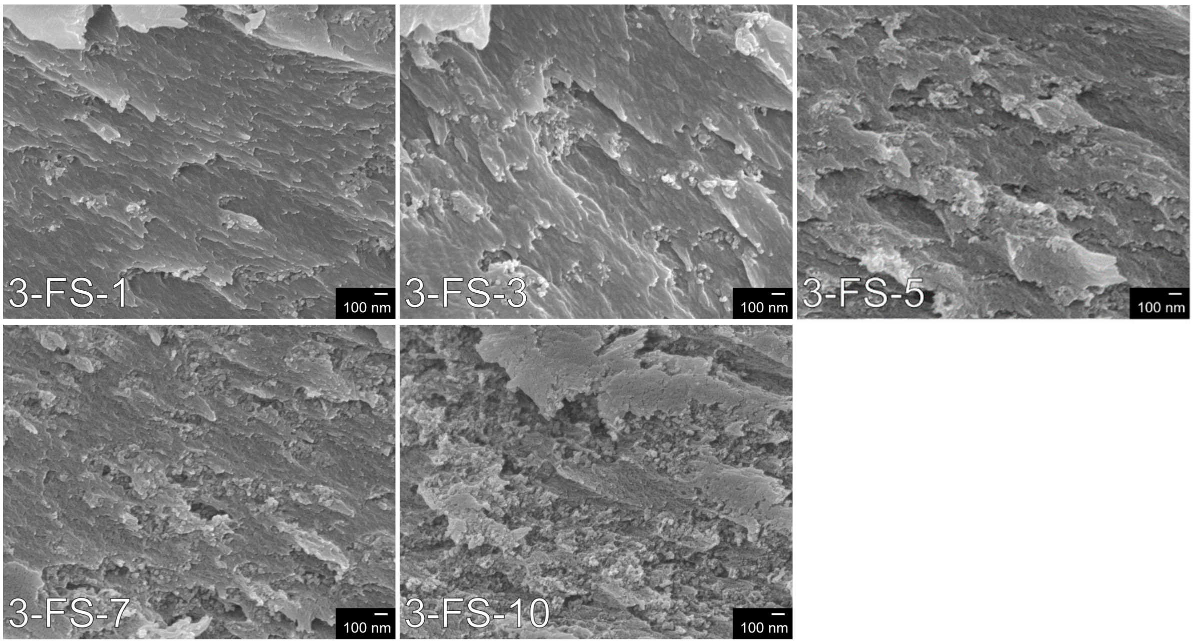
3-FS-1
100 nm
3-FS-3
100 nm
3-FS-5
100 nm
3-FS-7
100 nm
3-FS-10
100 nm

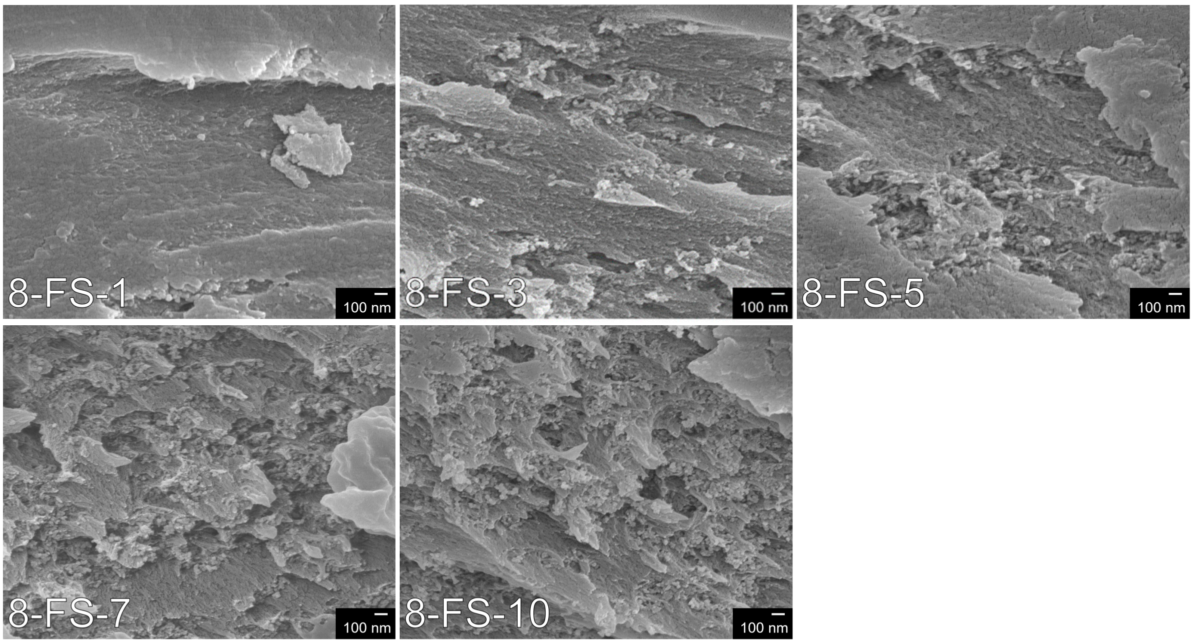
8-FS-1
100 nm
8-FS-3
100 nm
8-FS-5
100 nm
8-FS-7
100 nm
8-FS-10
100 nm

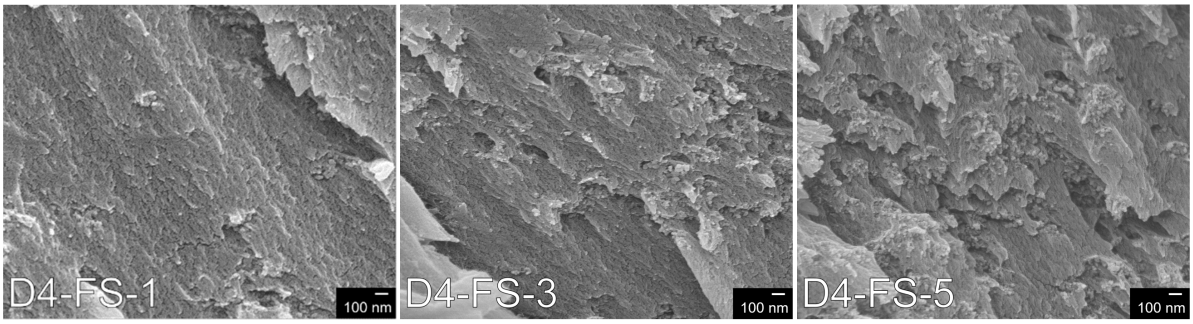
D4-FS-1
100 nm
D4-FS-3
100 nm
D4-FS-5
100 nm

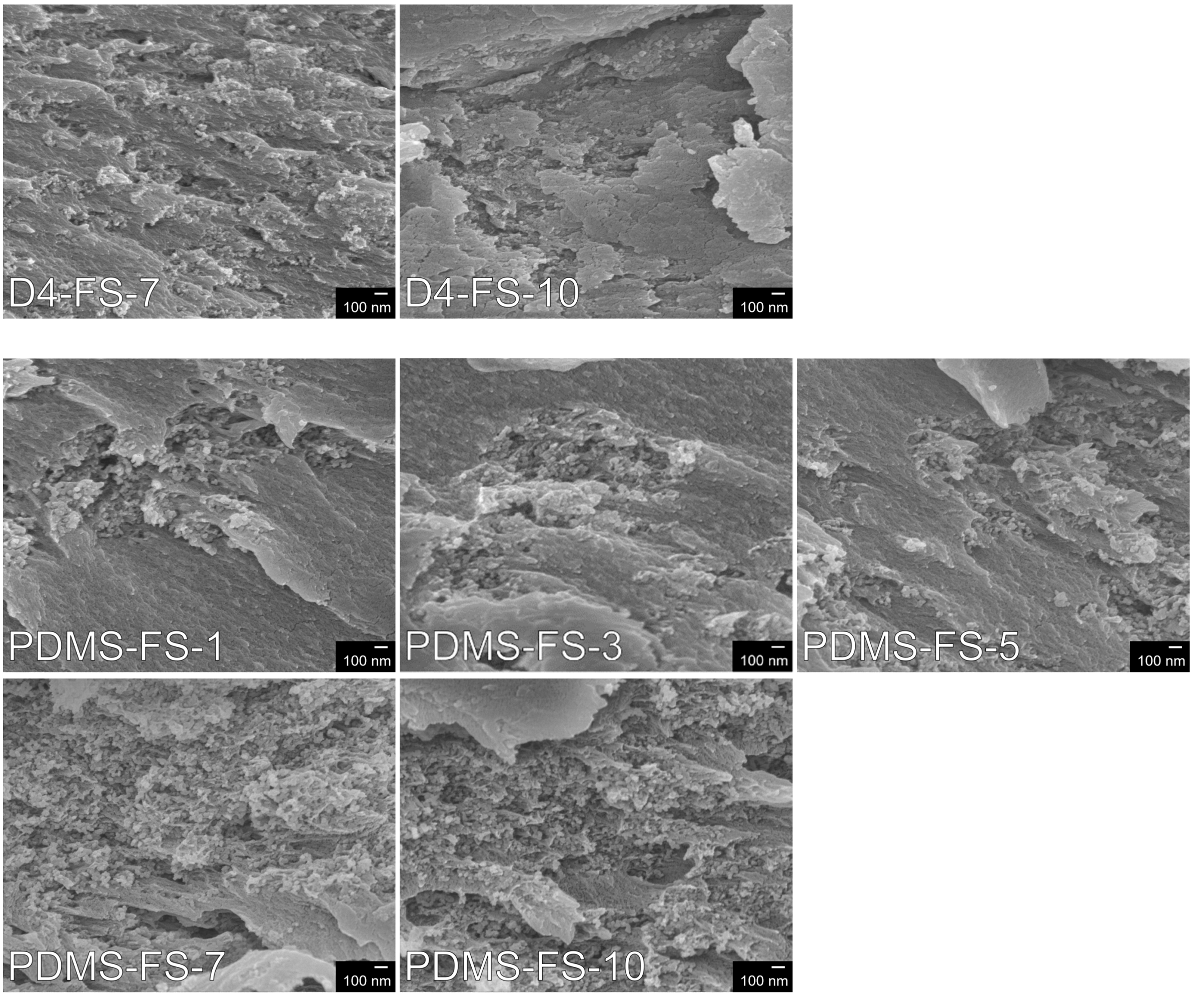

Figure S5. Cross-sectional SEM images of PI/FS composite films.

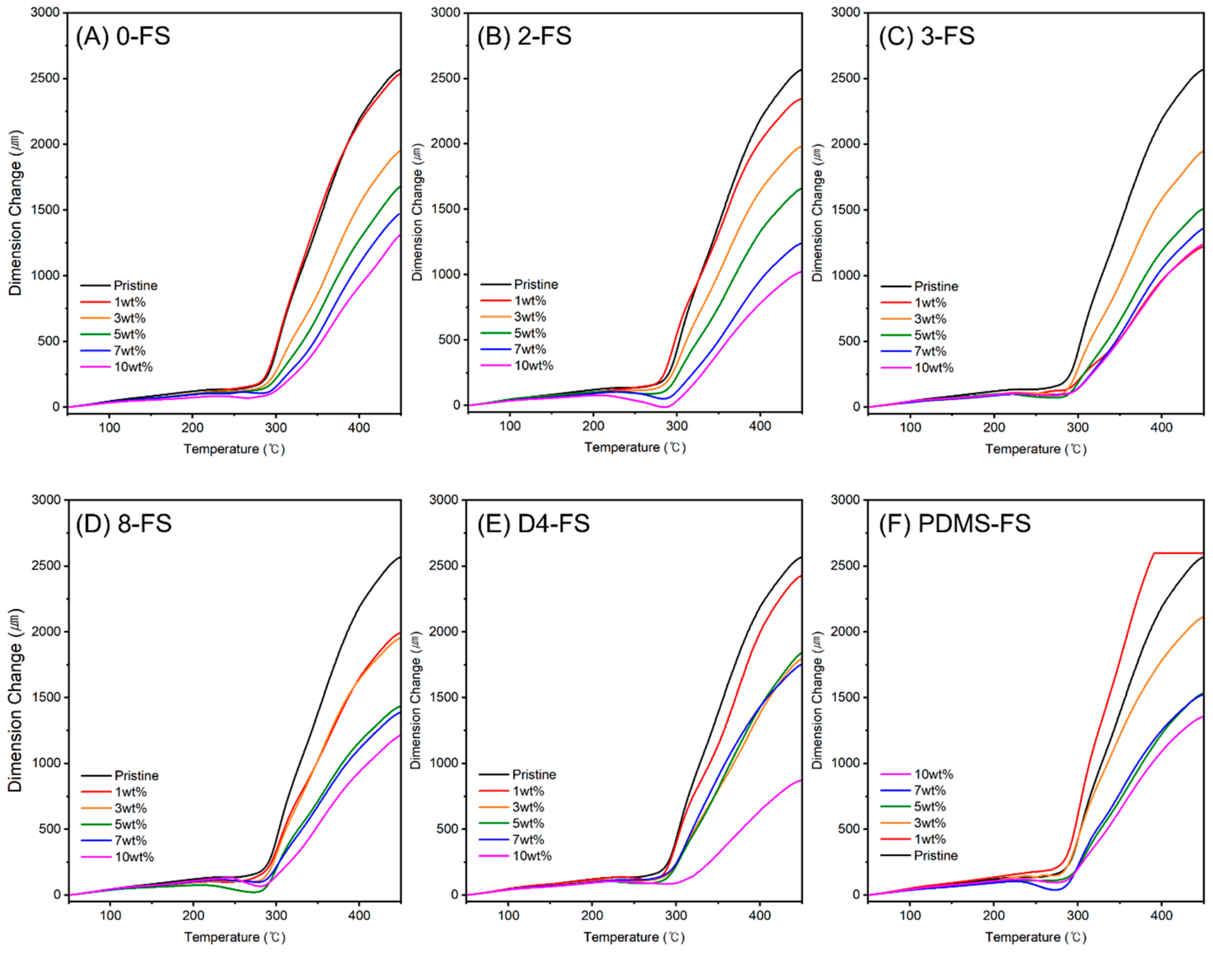

Figure S6. TMA curves of the PI/FS composite films containing (A) 0-, (B) 2-, (C) 3-, (D) 8-, (E) D4-, and (F) PDMS-FS.

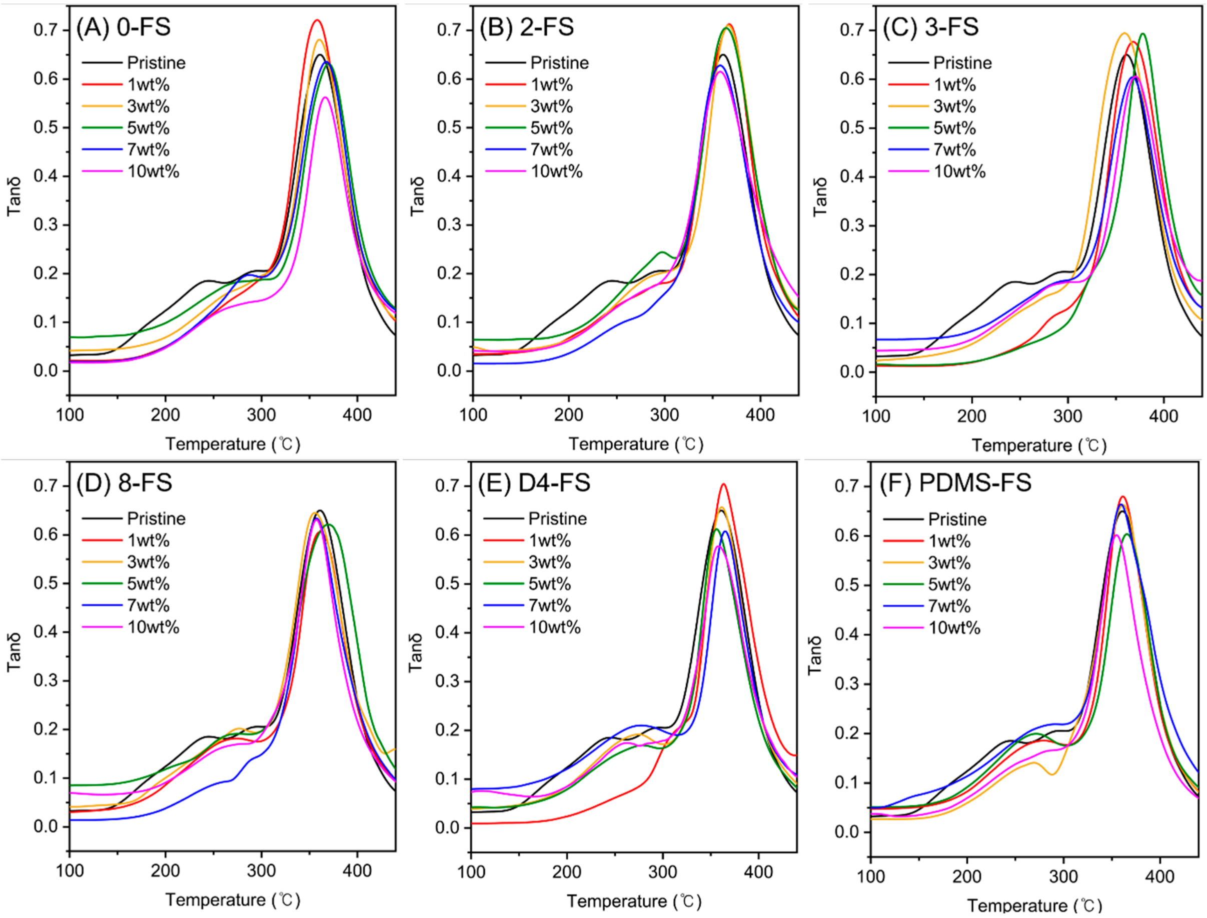

Figure S7. Tan δ curves of the PI/FS composite films containing (A) 0-, (B) 2-, (C) 3-, (D) 8-, (E) D4-, and (F) PDMS-FS.
